# Relationship between homoeologous regulatory and structural genes in allopolyploid genome – A case study in bread wheat

**DOI:** 10.1186/1471-2229-8-88

**Published:** 2008-08-13

**Authors:** Elena K Khlestkina, Marion S Röder, Elena A Salina

**Affiliations:** 1 Institute of Cytology and Genetics, Siberian Branch of the Russian Academy of Sciences, Lavrentjeva ave. 10, Novosibirsk, 630090, Russia; 2 Leibniz Institute of Plant Genetics and Crop Plant Research (IPK), Corrensstr. 3, D-06466 Gatersleben, Germany

## Abstract

**Background:**

The patterns of expression of homoeologous genes in hexaploid bread wheat have been intensively studied in recent years, but the interaction between structural genes and their homoeologous regulatory genes remained unclear. The question was as to whether, in an allopolyploid, this interaction is genome-specific, or whether regulation cuts across genomes. The aim of the present study was cloning, sequence analysis, mapping and expression analysis of *F3H *(flavanone 3-hydroxylase – one of the key enzymes in the plant flavonoid biosynthesis pathway) homoeologues in bread wheat and study of the interaction between *F3H *and their regulatory genes homoeologues – *Rc *(red coleoptiles).

**Results:**

PCR-based cloning of *F3H *sequences from hexaploid bread wheat (*Triticum aestivum *L.), a wild tetraploid wheat (*T. timopheevii*) and their putative diploid progenitors was employed to localize, physically map and analyse the expression of four distinct bread wheat *F3H *copies. Three of these form a homoeologous set, mapping to the chromosomes of homoeologous group 2; they are highly similar to one another at the structural and functional levels. However, the fourth copy is less homologous, and was not expressed in anthocyanin pigmented coleoptiles. The presence of dominant alleles at the *Rc-1 *homoeologous loci, which are responsible for anthocyanin pigmentation in the coleoptile, was correlated with *F3H *expression in pigmented coleoptiles. Each dominant *Rc-1 *allele affected the expression of the three *F3H *homoeologues equally, but the level of *F3H *expression was dependent on the identity of the dominant *Rc-1 *allele present. Thus, the homoeologous *Rc-1 *genes contribute more to functional divergence than do the structural *F3H *genes.

**Conclusion:**

The lack of any genome-specific relationship between *F3H-1 *and *Rc-1 *implies an integrative evolutionary process among the three diploid genomes, following the formation of hexaploid wheat. Regulatory genes probably contribute more to the functional divergence between the wheat genomes than do the structural genes themselves. This is in line with the growing consensus which suggests that although heritable morphological traits are determined by the expression of structural genes, it is the regulatory genes which are the prime determinants of allelic identity.

## Background

The flavonoid biosynthesis pathway is central to the formation of the phenolic compounds involved in many plant traits, including resistance to abiotic and biotic stresses [1-4]. One branch of the pathway is responsible for the generation of anthocyanin, which is present in various plant organs in most plant species, including the allohexaploid crop species, bread wheat (*Triticum aestivum *L.). Two major groups of anthocyanin pigmentation genes are present in wheat: the first includes *Rc-1*, *Pc-1*, *Pan-1*, *Plb-1 *and *Pls-1 *which encode the pigmentation in, respectively, the coleoptile, culm, anthers, leaf blades and leaf sheaths; while the second consists of *Pp *and *Ra*, which are expressed in, respectively, the pericarp and auricle [5]. The former genes are closely linked to one another on each of the short arms of the homoeologous group 7 chromosomes. An orthologue of maize gene *c1 *(which encodes a Myb-like transcriptional factor controlling tissue-specific anthocyanin biosynthesis [6]) was mapped earlier on each of the short arms of wheat homoeologous group 7 chromosomes, too [7] in positions highly comparable to those of *Rc-1 *(red coleoptile) genes [5,8]. Furthermore, it was shown that *c1*, when transferred to wheat, was able to induce anthocyanin pigmentation in non-pigmented wheat coleoptiles [9]. At the same time *Rc-1 *was shown to upregulate a number of wheat flavonoid biosynthesis pathway genes – *DFR *(dihydroflavonol-4-reductase), *ANS *(anthocyanidin synthase) and *UFGT *(UDPG flavonol 3-0-glucosyl transferase) [10,11]. Recognizing elements for *c1 *have also been identified in the promoter sequence of *Arabidopsis thaliana F3H *gene (flavanone 3-hydroxylase – one of the key enzymes involved in the biosynthesis of flavonoid compounds [12]), suggesting that *Rc-1 *can probably exert a regulatory role for wheat *F3H*, too. *F3H *orthologues have been isolated in barley and maize [13,14] as well as in a range of other plant species , but have yet to be described in wheat.

The patterns of expression of homoeologous genes in wheat have been intensively studied in recent years [15-21], but the interaction between structural genes and their homoeologous regulatory genes is unclear. The question remains as to whether, in an allopolyploid, this interaction is genome-specific, or whether regulation cuts across genomes. The *Rc-1 *and *F3H *genes are a suitable model to investigate just this issue, as the expression of *Rc-1 *generates a clear phenotype, and the latter are well-characterized at the molecular level. In this paper, we describe the cloning, sequence analysis, mapping and expression of *F3H *orthologues in bread wheat and its relatives, and the interaction between *F3H *and the *Rc-1 *homoeologues.

## Results

### Sequence analysis of *F3H *genes in wheat and its relatives

**Figure 1:**
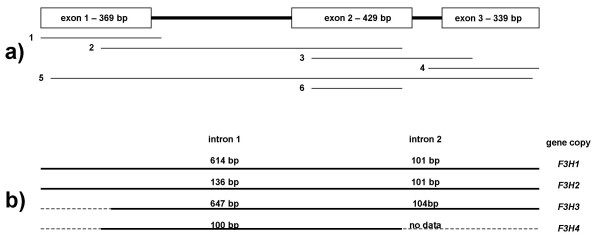
**The structure of wheat *F3H*.** Different gene segments referred in the paper text and tables are indicated in the figure part **(a)**, length of introns of the different *T. aestivum F3H *gene copies are indicated in part **(b)**; partial sequences are extended with dotted lines, whereas solid lines correspond to sequences cloned and analysed in the present study.

**Figure 2:**
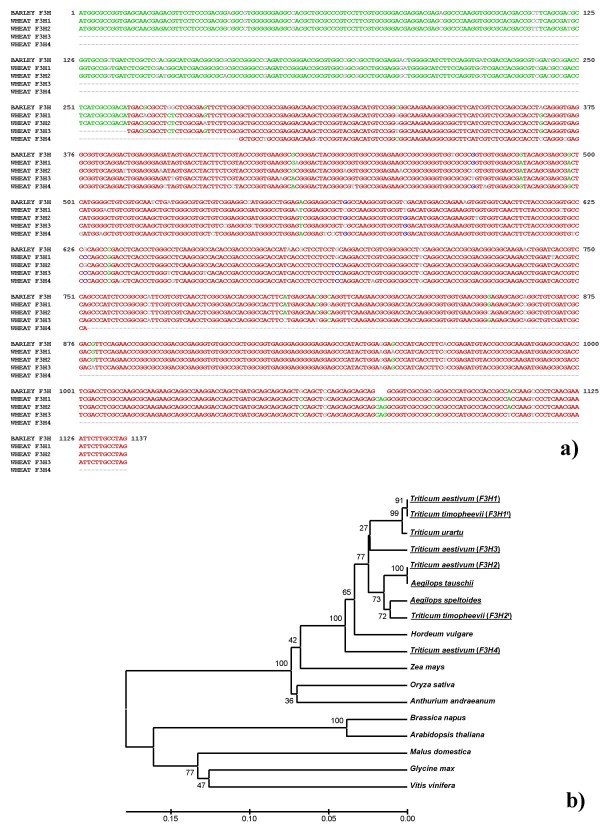
***F3H *****sequences comparison: (a) **alignment of complete coding sequences of barley *F3H *[13] and wheat *F3H1 *and *F3H2 *and partial wheat *F3H3 *and *F3H4 *copies cloned in the present study (introns are not included into alignment); **(b) **similarity of part of *F3H *exon 2 (specified as segment 6 in Figure 1) from various plant species – the species from which *F3H *copies were cloned and analysed in the present study are underlined, others were obtained from GenBank; for species with more than one *F3H *gene, each copy is identified by a number in parentheses.

**Figure 3:**
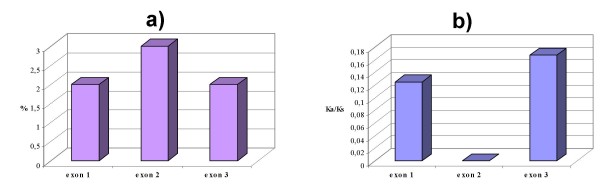
**Gene divergence between the hexaploid wheat A and D genome *F3H *gene copies: (a) **percentage of nucleotide substitutions in exons, **(b) **ratio of non-synonymous (Ka) to synonymous (Ks) nucleotide substitutions.

Nine *F3H *copies were isolated by PCR cloning from bread wheat (genome AABBDD), the tetraploid wild wheat *T. timopheevii *(AAGG) and the presumed diploid progenitors of the A, B/G and D genomes (A: *T. urartu*, B/G:*Ae. speltoides*, D: *Ae. tauschii*) (Table 1). Four of the copies were isolated from bread wheat. The length of the coding sequence, which was split into three exons, was 1137 bp, and the first intron varied in length among the homoeologues by some hundreds of base pairs (Figure 1). The sequence of the segments of the first intron of the bread wheat copies *F3H1*, *F3H2 *and *F3H3 *not affected by deletions/insertions shared over 80% homology, but the first intron of *F3H4 *was quite distinct. Sequence alignment of *T. aestivum F3H *sequences (coding regions) with barley *F3H *[13] is shown in Figure 2a. Sequence comparisons between exon 2 of the *Triticum *and *Aegilops F3H *genes (as well as other *F3H *sequences lodged in GenBank) are illustrated as dendrogram in Figure 2b. The *F3H4 *sequence departs significantly from that of the other *Triticum *and *Aegilops *copies (Figure 2). *T. aestivum F3H1 *and *T. timopheevii F3H1*^*t *^sequences are probably derived from the A genome, whereas *F3H2 *and *F3H2*^*t *^are suggested to belong to the genomes D and G, respectively (Figure 2b). *F3H3 *occupies an intermediate position between the two main *Triticum*-*Aegilops *clusters (Figure 2b). Patterns of sequence divergence across the structural region of wheat *F3H1 *and *F3H2 *suggest that the second exon is the most variable at the nucleotide level, but is most well conserved at the amino acid level (Figure 3, Table 2). Exon 2, intron 2 and the beginning of exon 3 (Segment 3, see Figure 1) were re-sequenced from a panel of seven diverse bread wheat genotypes, but no intraspecific variation was detected.

**Table 1:** Length, Genbank accession numbers and chromosome locations for *F3H *nucleotide sequences determined in the present study.

Species, gene	Length in base pairs (gene segment specification according Figure 1)	Genbank accession number	Identical wheat ESTs*	Chromosome location
*T. aestivum, F3H1*	1852, complete structural part of gene (Segments 1+2+3+4)	EF463100	BG262227	2A
*T. aestivum, F3H2*	1374, complete structural part of gene (Segments 1+4+5)	DQ233636	BJ237068 BJ242608	2D
*T. aestivum, F3H3*	1626, partial (Segments 2+3+4)	EU402957	BQ240612 BG262749 CA705431	2B
*T. aestivum, F3H4*	562, partial (Segment 2)	EU402958	BE414777	2B
*T. timopheevii, F3H1*^*t*^	542, partial (Segment 3)	EU402959	BG262227	2A
*T. timopheevii, F3H2*^*t*^	539, partial (Segment 3)	EU402960	-	2G
*T. urartu, F3H*	542, partial (Segment 3)	EU402961	BG262227	Suggested 2A
*Ae. speltoides, F3H*	542, partial (Segment 3)	EU402963	-	Suggested 2S
*Ae. tauschii, F3H*	1326, partial (Segment 5)	DQ233637	BJ237068 BJ242608	Suggested 2D

*Correspondence between *F3H *copies and ESTs was first determined based on identity at gene copy-specific sights, and then was confirmed by whole sequences comparison (see Figure 10).

**Table 2:** Sequence homology and divergence among *F3H1 *and *F3H2 *genes.

Part of gene	Length: *F3H1*/*F3H2 *(in bp)	Nucleotide sequences homology (%)	Ka/Ks*
Exon1	369/369	98	0.125
Intron 1	614/136	There are two major deletion regions, other segments have over 90% homology	-
Exon 2	429/429	97	0.000
Intron 2	101/101	97	-
Exon 3	339/339	98	0.167

*Ka – non-synonymous nucleotide substitutions, Ks – synonymous nucleotide substitutions.

### Chromosomal assignment and physical mapping of *F3H *genes in hexaploid wheat

**Figure 4:**
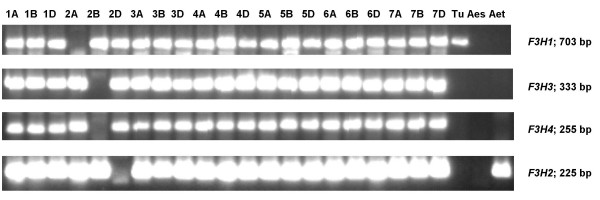
**PCR profiles of the 'Chinese Spring' nulli-tetrasomic lines and the diploid donors of hexaploid wheat, amplified with *F3H *copy-specific primers.** The length of the PCR products is given in base pairs to the right. Designations '1A', '1B' etc. correspond to 'nulli' chromosome in the certain nulli-tetrasomic line; 'Tu' – *T. urartu*, 'Aes' – *Ae. speltoides*, 'Aet' – *Ae. tauschii*.

**Figure 5:**
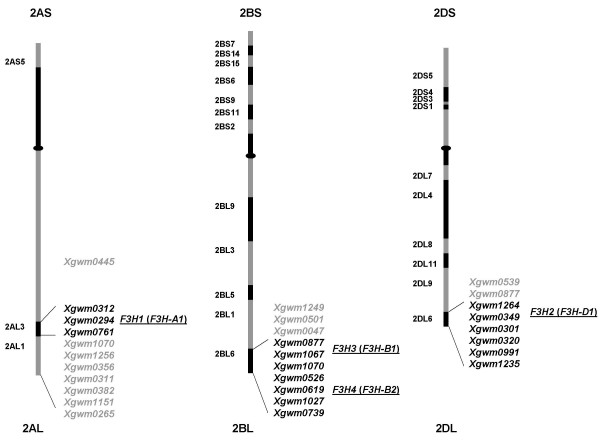
**Physical mapping of *F3H *loci in bread wheat performed using subset of *T. aestivum *cv. 'Chinese Spring' homoeologous group 2 chromosomes deletion lines.** Microsatellite markers (*Xgwm*) designations are given to the right from each chromosome scheme, chromosome bin names are indicated to the left.

**Figure 6:**
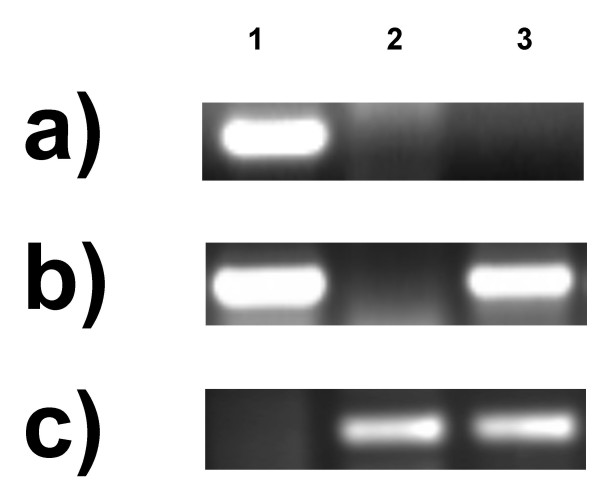
PCR profiles of 'Saratovskaya 29' (1), *T. timopheevii *(2) and 'Saratovskaya 29'x *T. timopheevii *introgression line 842 (3), amplified with gene copy-specific primers for *T. aestivum F3H3 *(a) and *F3H4 *(b) and *T. timopheevii F3H2*^*t *^(c).

Primer pairs amplifying specifically fragments from individual *F3H *copies (referred further as "gene copy-specific primer pairs") were designed and used in PCR analysis of 'Chinese Spring' nulli-tetrasomic lines. It was shown that *F3H1 *and *2 *are on, respectively, chromosomes 2A and 2D, while *3 *and *4 *both map to chromosome 2B (Table 1, Figure 4). A deletion line analysis was then used to define the intra-chromosomal location of *F3H1 *to the sub-terminal bin (2AL3) of chromosome 2AL, both *F3H3 *and *F3H4 *to the terminal bin (2BL6) of chromosome 2BL, and *F3H2 *to the terminal bin (2DL6) of chromosome 2DL (Figure 5). Since the location of *F3H3 *and *F3H4 *could not be distinguished by this method, an introgression line derived from the cross *T. aestivum *× *T. timopheevii*, which contains a 2BL/2GL breakpoint within chromosome bin 2BL6 between the microsatellite loci *Xgwm1067 *and *Xgwm0526 *[22], was used to show that *F3H3 *and *-4 *are discrete loci (Figure 6a and 6b, respectively). *F3H3 *lies proximal to the to the 2BL/2GL breakpoint, whereas *F3H4 *location is distal. A specific PCR assay for the *T. timopheevii F3H2*^*t *^sequence (Figure 6c) proved that it, like *T. aestivum F3H3*, too lies proximal to the 2BL/2GL breakpoint, thus suggesting that these two loci, along with *F3H1 *and *F3H2*, belong to an *F3H *homoeoallelic series, whereas *F3H4 *appears to be a non-homoelogous duplication. Accordingly, the genes were re-designated *F3H-A1 *(*F3H1*), *F3H-B1 *(*F3H3*), *F3H-D1 *(*F3H2*), *F3H-G1 *(*F3H2*^*t*^) and *F3H-B2 *(*F3H4*).

### Expression analysis of *F3H *in lines with and without pigmented coleoptiles

**Figure 7:**
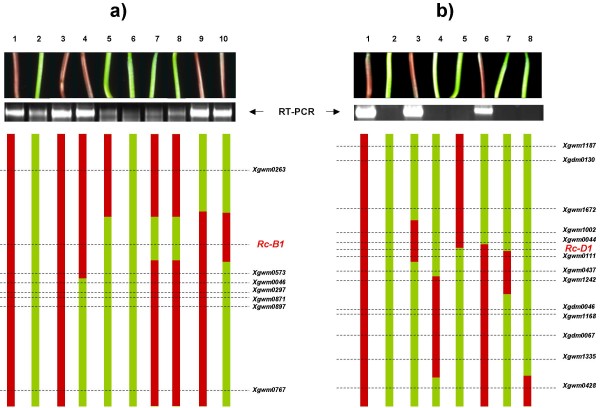
**RT-PCR analysis of total F3H expression in four day old seedlings of **(a) **'Chinese Spring' ('Hope' 7B) (1), 'TRI 2732' (2) and progeny of the cross 'Chinese Spring' ('Hope' 7B) × 'TRI 2732' (3–10); **(b) **substitution 'Chinese Spring' (*Ae. tauschii *7D) (1), 'Chinese Spring' (2) and the 'Chinese Spring'/*Ae. tauschii *7D introgression lines (3–8).** Anthocyanin pigmentation in coleoptiles of the corresponding lines is shown above, whereas the status of chromosomes 7B **(a) **or 7D **(b) **of each line is indicated in the lower part of the panel.

**Figure 8:**
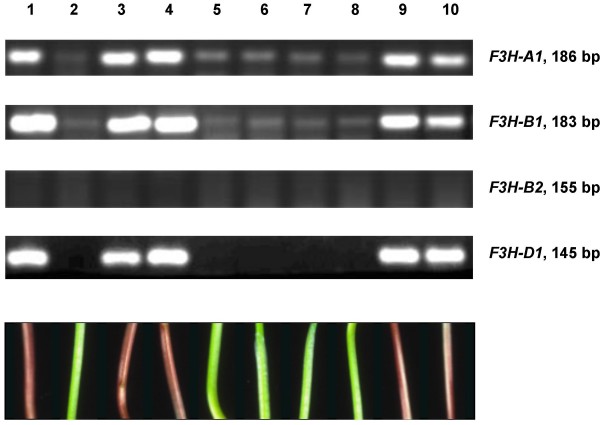
***F3H *****copy-specific RT-PCR analysis from four day old seedlings of 'Chinese Spring' ('Hope' 7B) (1), 'TRI 2732' (2) and progeny of the cross 'Chinese Spring' ('Hope' 7B) × 'TRI 2732' (3–10).** Anthocyanin pigmentation in coleoptiles of the corresponding lines is shown below. The length of the RT-PCR products is given in base pairs to the right.

To explore the role of the *Rc-1 *(red coleoptile) genes as regulators for F3H expression, eight progeny from the cross 'Chinese Spring' ('Hope' 7B) × 'TRI 2732', along with a set of six different chromosome 7D introgression lines of *Ae. tauschii *into 'Chinese Spring', varying with respect to the dominant allele at either *Rc-B1 *or *Rc-D1*, were subjected to RT-PCR analysis from cDNA derived from four day old seedlings. The parental genotypes with pigmented coleoptiles ('Chinese Spring' ('Hope' 7B) and 'Chinese Spring (*Ae. tauschii *7D) both showed a high level of F3H expression, whereas those with non-pigmented coleoptiles showed either little ('TRI 2732') or none ('Chinese Spring') (Figure 7). When this result was compared with the microsatellite-based genotype of the lines [8,23], the regulator of F3H expression on chromosome 7B was mapped between *Xgwm0263 *and *Xgwm0573*, co-segregating with *Rc-B1 *(Figure 7a); similarly, the equivalent locus on chromosome 7D co-segregated with *Rc-D1 *within the genetic interval *Xgwm0044 *and *Xgwm0111 *(Figure 7b). RT-PCR was also used to study contribution of single genes *F3H-A1*, *F3H-B1*, *F3H-B2 *and *F3H-D1 *to total F3H expression. It was shown that *F3H-B2 *is not expressed whether or not the coleoptiles are pigmented (Figure 8). In contrast, *F3H-A1*, *F3H-B1 *and *F3H-D1 *were actively expressed in lines with pigmented coleoptiles ('Chinese Spring' ('Hope' 7B) and respective recombinant lines; Figure 8, lines 1, 3, 4, 9, 10), whereas those with non-pigmented coleoptiles ('TRI 2732' and respective recombinant lines) showed a low level of expression of only *F3H-A1 *and *F3H-B1 *(Figure 8: faint bands in lines 2, 5–8, respectively).

### Temporal pattern and the genome specificity of *F3H *expression

**Figure 9:**
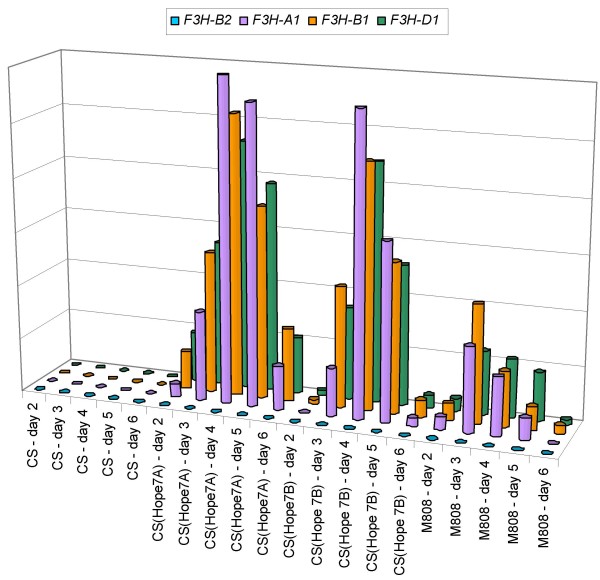
Quantitative RT-PCR analysis with respect to the various copies of *F3H *in 'Chinese Spring' (CS), 'Chinese Spring' ('Hope' 7A), 'Chinese Spring' ('Hope' 7B) and 'Mironovskaya 808' (M808).

To investigate the possibility of more subtle differences between expression levels of the *F3H *homoeologues in presence of particular alleles of *Rc-1*, quantitative RT-PCR was applied to a set of cDNAs sampled from two to six day old seedlings (Figure 9). The test genotypes were 'Chinese Spring' ('Hope' 7A) [*Rc-A1b*], 'Chinese Spring' ('Hope' 7B) [*Rc-B1b*] and cv. 'Mironovskaya 808' [*Rc-D1b*], along with the control 'Chinese Spring' which carries the non-pigmented alleles at all three *Rc-1 *loci. In the latter, none of the *F3H *copies was expressed at any time during the sampling period. *F3H-B2 *was not expressed in any of three test line seedlings, but *F3H-A1*, *F3H-B1 *and *F3H-D1 *were all expressed in these lines. No within genotype significant difference (p = 0.05) in the expression level of the three homoeologues could be detected at any of the sampling times (Table 3). However, the overall level of F3H expression differed very significantly between each pair of lines (Table 4). The level was lowest in 'Mironovskaya 808' and highest in 'Chinese Spring' ('Hope' 7A). The highest expression level in 'Mironovskaya 808' was reached three days after germination, while in 'Chinese Spring' ('Hope' 7A) and 'Chinese Spring' ('Hope' 7B), the maximum was detected on the fourth day. In 'Chinese Spring' ('Hope' 7B), expression started later and declined more rapidly than in 'Chinese Spring' ('Hope' 7A). The delayed start and lower total level of expression in 'Chinese Spring' ('Hope' 7B) was consistent with the observed temporal development of pigmentation in the coleoptiles. Overall, therefore, each *Rc-1 *gene appeared to regulate the expression of the three *F3H *homoeologues equally, but the level of *F3H *expression was dependent on the identity of the dominant *Rc-1 *allele present.

**Table 3:** T-values for expression levels of different *F3H *homoeologues in coleoptiles (p = 0.05 for all presented values).

	*F3H-A1 vs F3H-B1*	*F3H-A1 vs F3H-D1*	*F3H-D1 vs F3H-B1*
'Chinese Spring' ('Hope' 7A)	0.28	0.40	0.40
'Chinese Spring' ('Hope' 7B)	0.04	0.48	1.92
'Mironovskaya 808'	1.39	0.27	0.52

**Table 4:** T-values for F3H expression in different wheat genotypes.

Genotypes compared	'Chinese Spring' ('Hope' 7A) *vs *'Chinese Spring' ('Hope' 7B)	'Chinese Spring' ('Hope' 7A) *vs *'Mironovskaya 808'	'Chinese Spring' ('Hope' 7B) *vs *'Mironovskaya 808'
T	6.17	4.29	2.76
P >	0.999	0.999	0.95

## Discussion

### Cloning and analysis of *F3H *sequences

**Figure 10:**
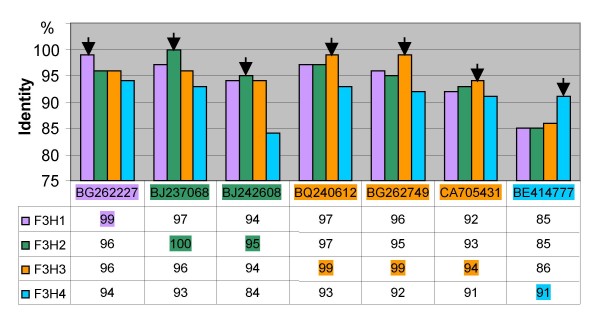
**Comparison of *T. aestivum F3H *copies *vs *homologous wheat ESTs.** The highest identity value for each EST is indicated with black arrow.

*F3H *genes have been isolated from barley, maize and *Arabidopsis thaliana *[13,14,24] as well as from a range of other plant species . In wheat, only one single partial *F3H *sequence has been published to date [11]. The relationship between the wheat and *Aegilops *sp. *F3H *sequences reported here (with the exception of *F3H-B2*) and those lodged in GenBank (Figure 2) is consistent with standard taxonomic treatment [25] and with known phylogenies within the *Triticum*/*Aegilops *complex [26]. The *F3H *sequences of diploid progenitors of wheat were useful for the genome assignment of the homoeologous gene copies in polyploid wheat. The substantial structural divergence between *F3H-B2 *and that of three *F3H-1 *homoeologues is accompanied by a functional difference. The lack of *F3H-B2 *expression in pigmented coleoptiles does not reflect its complete non-functionality, since a highly identical root EST has been reported (Table 1, Figure 10). The presence of two B genome copies of *F3H *is not a particularly unusual result, as *F3H *copy number in diploids varies from one [13,14,24] to two [27,28]. Silent divergence (Ka/Ks) appears to be homogeneously distributed throughout the coding region of *Arabidopsis thaliana F3H*, being rarest in the second, and most frequent in the third exon [29]. A similar pattern applies to the wheat A and D genome *F3H *homoeologues (Figure 3, Table 2).

A PCR-based cloning approach has been used to clone other flavonoid biosynthesis pathway genes in hexaploid wheat (Table 5), whereas in barley and other diploid species they have been isolated from cDNA libraries [13,30]. It has recently become clear that not all members of a homoeologous series in wheat are co-expressed [16,18,31], so the genomic PCR-based cloning approach is probably the more preferable strategy to capture a full set of homoeologues. Although PCR-based cloning has some disadvantages when applied in an allopolyploid (specifically in the generation of PCR chimeras – however, this problem can usually be overcome by the cloning and sequencing of several replicates), it is an effective strategy for the design of gene copy-specific primers, the chromosomal localization of genes and expression analysis.

**Table 5:** Previously characterised flavonoid biosynthesis pathway genes in wheat.

	Gene cloning			Mapping
		
Enzyme	Cloning approach	Number of cloned copies	Genbank accessions; references	Chromosome location; references
PAL – phenylalanine ammonialyase	Isolation from genomic library	2 complete	X99705[41,42]	3A, 3B, 3D, 6A, 6B, 6D [7]
CHS – chalcone synthase	PCR-based cloning	4 complete	AY286093, AY286095, AY286096, AY286097[43]	1A, 1B, 1D, 2A, 2B, 2D [7]
CHI – chalcone-flavanone isomerase	PCR-based cloning	1 partial	AB187026[11]	5A, 5B, 5D [7]
F3H – flavanone 3-hydroxylase	PCR-based cloning	1 partial	AB187027[11]	-
F3'5'H – flavonoid 3',5'-hydroxylase	PCR-based cloning	1 partial	AY519468[43]	-
DFR – dihydroflavonol-4-reductase	PCR-based cloning	3 complete	AB162138, AB162139, AB162140[44]	3AL, 3BL, 3DL [44,45]
ANS – anthocyanidin synthase	Not described	5 complete	AB247917, AB247918, AB247919, AB247920, AB247921[46]	6AS (2 copies), 6BS (2 copies), 6DS [46]
FMT – flavonoid 7-O-methyltransferase	-	-	-	1A, 1B, 1D [7]

### Expression of the three homoeologous *F3H *loci in lines with and without pigmented coleoptiles

The patterns of expression of flavanone 3-hydroxylase in lines with and without pigmented coleoptiles indicated that *Rc-B1 *and *Rc-D1 *are coincident with the genes regulating its expression (Figure 7). This is in accordance with the suggestion that *Rc-1 *genes exert a regulatory role for *F3H *genes, which could be made on the base of combined results obtained earlier [5-9,12]. The patterns of temporal expression among the *F3H *homoeologues in the presence of different dominant *Rc-1 *alleles allowed for an examination as to whether, in an allopolyploid context, there are any genome-specific relationships between the structural and regulatory genes. No such relationship was apparent, since in pigmented coleoptiles, *F3H-A1*, *F3H-B1 *and *F3H-D1 *were all expressed at a similar level (Figure 9). Many sets of wheat homoeologous genes are known to be equally expressed in this way [16,19,21], but in others, the expression of one or more members may be either completely [16,18,31] or partially [15,20,21] suppressed. Generally, when *F3H *homoeologues are expressed actively (as in pigmented coleoptiles), then they are expressed equally, but where overall *F3H *transcription level is low, then selective expression of *F3H *homoeologues could be observed (i.e. *F3H-A1 *and *F3H-B1 *were expressed in the green coleoptiles of 'TRI 2732', but *F3H-D1 *was not; Figure 8). These outcomes are consistent with the activity-selectivity principle [32] acting at the transcriptional level.

### Functional difference between homoeologous *Rc-1 *genes

Whereas each dominant *Rc-1 *allele affects the expression of each of the three *F3H *homoeologues equally, overall F3H expression was dependent on the identity of which dominant *Rc-1 *allele was present (Figure 9). This difference was observed not only at specific time points, but also from the total amounts of *F3H *mRNA produced over the period of coleoptile pigmentation. The delayed start of expression and the lesser level of transcript present in 'Chinese Spring' ('Hope' 7B) compared to 'Chinese Spring' ('Hope' 7A) was consistent with the observed accumulation of pigmentation in the coleoptile, both in the present experiments and in those reported earlier [33]. In order to test for background effects on *F3H *expression or variability within transcriptional factors encoded by dominant *Rc-1 *alleles in other genotypes, it would be of interest to investigate the extent to which the profiles of *F3H *expression of 'Chinese Spring' ('Hope' 7A), 'Chinese Spring' ('Hope' 7B) and 'Mironovskaya 808' are typical, i.e. for instance to compare profile of 'Mironovskaya 808' to those of some other varieties carrying the same dominant allele (*Rc-D1*).

## Conclusion

There are at least four flavanone 3-hydroxylase gene copies in the hexaploid genome of bread wheat, three of which are the homoeologues on chromosomes 2AL, 2BL and 2DL, highly similar at structural and functional level, while the fourth one represents a distinct non-homoeologous copy on chromosome 2BL with suppressed expression in red coleoptiles.

Expression of the F3H homoeologues (*F3H-1*) in wheat coleoptiles is determined by the presence of dominant alleles in *Rc-1 *(red coleoptiles) loci. *Rc-1 *and *F3H-1 *genes represent a suitable model to investigate relationship between homoeologous regulatory and homoeologous structural genes in allopolyploid wheat genome (which have never been studied before). The lack of any genome-specific relationship between *F3H-1 *and *Rc-1 *observed in the present study implies an integrative evolutionary process among the three diploid genomes, following the formation of hexaploid wheat.

Furthermore, based on *F3H *expression analysis it was observed for the first time that activity-selectivity principle [32] acts at the transcriptional level.

Our general conclusion is that regulatory genes probably contribute more to the functional divergence between the wheat genomes than do the structural genes themselves. This is in line with the growing consensus which suggests that although heritable morphological traits are determined by the expression of structural genes, it is the regulatory genes which are the prime determinants of allelic identity.

## Methods

### Plant materials and RNA extraction

The bread wheat cultivars 'Chinese Spring', 'Opata', 'Flair', 'Prinz', 'Golubka', 'Novosibirskaya 67', the synthetic hexaploid wheat 'W7984', tetraploid *T. timopheevii *k-38555 (AAGG) and the diploids *T. urartu *TMU06 (AA), *Aegilops speltoides *TS01 (SS) and *Ae. tauschii *TQ17 (DD) were used for PCR-based cloning. The complete set of 'Chinese Spring' nulli-tetrasomic lines [34], a subset of homoeologous group 2 chromosome deletion lines [35], introgression line 842 derived from the cross *T. aestivum *cv. 'Saratovskaya 29' × *T. timopheevii *[22] were exploited to establish chromosome bin locations. Eight progeny from the cross 'Chinese Spring' ('Hope' 7B) × 'TRI 2732' [8] and a set of six homozygous lines each containing a different chromosome 7D segment derived from *Ae. tauschii *in a 'Chinese Spring' background [23] were used for RT-PCR. Quantitative examination of *F3H *expression was measured in 'Chinese Spring' and 'Mironovskaya 808' and the single chromosome substitution lines 'Chinese Spring' ('Hope' 7A) and 'Chinese Spring' ('Hope' 7B). DNA was extracted from seven day old seedlings following the procedure described earlier [36]. RNA was extracted from seedlings grown at 20°C under a 12 h day/12 h night regime using the QIAGEN  Plant Rneasy Kit, followed by DNAse treatment. For RT-PCR, RNA was extracted on the fourth day after germination. For quantitative RT-PCR, RNA was extracted every 24 h from two to six day old seedlings.

### PCR-based cloning and sequence analysis

The barley *F3H *cDNA sequence [13] was aligned with matching wheat ESTs lodged in , employing Multalin v5.4.1 (using absolute alignment score with gap value of 12 and gap length value of 2) [37]. Sets of primers flanking various *F3H *gene segments were designed using OLIGO software (Table 6) [38], with one primer pair as described earlier [11]. PCR reaction mixtures (50 μl) contained 50 ng template, 67 mM Tris HCl pH8.8, 1.8 mM MgCl_2_, 0.01% Tween 20, 18 mM (NH_4_)_2_SO_4_, 0.2 mM dNTP, 0.25 mM each primer and 1 U Taq DNA polymerase. PCR amplifications began with a 94°C/5 min incubation, followed by 45 cycles of 94°C/1 min, 60°C/2 min, 72°C/2 min, and a final extension of 72°C/10 min. PCR fragments were recovered from 1% agarose gels, purified using a QIAGEN MinElute Gel Extraction Kit, and cloned with a QIAGEN PCR Cloning Kit. Between five and ten clones per each primer combination per diploid genome were sequenced in both directions to eliminate PCR and sequencing errors or PCR-generated chimeras. Sequencing was effected using an ABI PRISM Dye Terminator Cycle Sequencing ready reaction kit ("Perkin Elmer") with pUC/M13 forward and reverse primers. Full-(or partial) length sequences of various *F3H *gene copies were constructed from overlapping sequences. Cluster analysis was performed on MEGA v3.1 software [39] using the UPGMA (unweighted pair-group method with arithmetic average) algorithm and 500 bootstrap trials.

**Table 6:** Primers designed to amplify wheat *F3H *for cloning, chromosomal localization and for expression analysis.

Purpose	Gene	Gene segment specification (according Figure 1) or former gene name	DNA/cDNA-derived PCR product length (bp)	Forward primers	Reverse primers
PCR-based cloning	*F3H*	Segment 1	variable	atggcgccggtgagcaac	tttacgtggcatggcatgcat
	*F3H*	Segment 2	variable	atgacgcgcctctctcgcg	tggacggtgatccaggtcttg
	*F3H*	Segment 3	variable	[11]	[11]
	*F3H*	Segment 4	variable	tctcgatcgatcgaccaccaa	ctaggcaagaatttcgttgaggg
	*F3H*	Segment 5	variable	ccggtgagcaacgagacgttc	ggcaagaatttcgttgagggg
Chromosomal assignment and	*F3H*-*A*1	*T. aestivum F3H1*	703/-	gccacctgcaggtatacacgcat	ccaccgcccgtagtccct
physical mapping	*F3H*-*B*1	*T. aestivum F3H3*	333/-	gcgtgctgtccgaggcgc	cgatcgatcgattaaggatt
	*F3H*-*B2*	*T. aestivum F3H4*	255/155	gctgcctgccgaggacaagg	aacgcccgtagtcccgtgcc
	*F3H*-*D*1	*T. aestivum F3H2*	225/-	gccacctgcaggtacccacacat	ccacctcccgtagtcccg
	*F3H*-*G*1	*T. timopheevii F3H2*^*t*^	371/-	acgactcatggggctgtca	caattggtggtcgatcgatcag
qRT-PCR, RT-PCR	*F3H*-*A*1	*T. aestivum F3H1*	800/186	atgacacgcctctctcgcg	ccaccgcccgtagtccct
	*F3H*-*B*1	*T. aestivum F3H3*	830/183	tgacgcgcctctctcgcgag	accgcccgtagtcccgtgct
	*F3H*-*B2*	*T. aestivum F3H4*	255/155	gctgcctgccgaggacaagg	aacgcccgtagtcccgtgcc
	*F3H*-*D*1	*T. aestivum F3H2*	281/145	atcgtctccagccacctgcag	cgctgtatcgctccaccacg

### Chromosomal assignment and physical mapping of F3H

Specific primer pairs were designed to amplify each wheat *F3H *copy (Table 6). To obtain a unique amplification product, the 3' end of at least one of the two primers matched the copy-specific sequence. A touchdown PCR protocol was used to amplify from templates of the 'Chinese Spring' nulli-tetrasomic and deletion lines and the *T. aestivum *× *T. timopheevii *introgression line 842 in 20 μl reactions by applying a denaturing step of 94°C/2 min, 13 cycles of 94°C/15 s, 65°C/30 s (decreasing by 0.7°C/cycle), 72°C/45 s, 24 cycles of 94°C/15 s, 56/30 s, 72°C/45 s; and a final extension of 72°C/5 min. The specificity of the amplifications was confirmed by cloning and sequencing of the PCR product from 'Chinese Spring'. The microsatellite analysis of the 'Chinese Spring' deletion lines was performed using procedures described earlier [40]. The microsatellite genotypic data of the *T. aestivum *× *T. timopheevii *introgression line 842 have been published [22].

### RT-PCR and qRT-PCR

Single-stranded cDNA was synthesized from 1 mg total RNA using a (dT)_15 _primer and the QIAGEN Omniscript Reverse Transcription kit in a 20 μl reaction mixture. RT-PCR was performed with *F3H *primers published earlier [11] or with *F3H *gene copy-specific primers (Table 6). The standardization of cDNA template was performed using ubiquitin (UBC) primers [11]. PCR products were separated by 2% agarose gel electrophoresis. *F3H *gene copy-specific primers were also applied for qRT-PCR, which used a QIAGEN QuantiTect SYBR Green kit. UBC and GAPDH primers were used to standardize the cDNA template. The amplifications were performed in an Applied Biosystems 7900 HT fast real time PCR system. Pre-determined amounts of cloned cDNA were used to generate standard curves. Each sample was run in three replicates. The specificity of the qRT-PCR products was confirmed by 2% agarose gel electrophoresis. Statistical significance of differences in *F3H *expression level either between *F3H *homoeologues or between different genotypes was assessed by Student's t-test for matched pairs. When *F3H *homoeologues were compared, T-values were calculated for each pair (*F3H-A1 vs F3H-B1*, etc.) in each genotype (Table 3), and 'matched pairs' were represented by expression level values obtained for respective pair of *F3H *homoeologues at the same day in the same genotype. When comparison was made between genotypes, T-values were calculated for each pair of genotypes ('Chinese Spring' ('Hope' 7A) *vs *'Chinese Spring' ('Hope' 7B), etc.; Table 4), and 'matched pairs' were represented by expression level values obtained in respective pair of genotypes at the same day for the same *F3H *gene copy.

### Accession numbers for sequence data

GenBank: EF463100, EU402957, EU402958, DQ233636, EU402959, EU402960, EU402961, EU402963, DQ233637.

## Authors' contributions

EKK carried out the molecular genetic studies, sequence alignment, primer design and statistical analysis, she conceived of the study, participated in its design and drafted the manuscript. MSR and EAS coordinated the study, contributed to its conception and design, to interpretation of data and to revising the manuscript critically. All authors read and approved the final manuscript.
